# Regional and socio-economic disparity in use of insecticide-treated nets to prevent malaria among pregnant women in Kenya

**DOI:** 10.1093/inthealth/ihac024

**Published:** 2022-04-29

**Authors:** Werissaw Haileselassie, Mizan Habtemichael, Ruth Adam, Jemal Haidar, Randy E David, Ayele Belachew, Abenet Tafesse Mengesha, Cristian Koepfli, Wakgari Deressa, Daniel M Parker, Nigussie Assefa Kassaw

**Affiliations:** School of Public Health, College of Health Sciences, Addis Ababa University, Addis Ababa, Ethiopia; School of Public Health, College of Health Sciences, Addis Ababa University, Addis Ababa, Ethiopia; School of Public Health, College of Health Sciences, Addis Ababa University, Addis Ababa, Ethiopia; School of Public Health, College of Health Sciences, Addis Ababa University, Addis Ababa, Ethiopia; Program in Public Health, College of Health Sciences, University of California at Irvine, Irvine, CA 92697, USA; School of Public Health, College of Health Sciences, Addis Ababa University, Addis Ababa, Ethiopia; School of Medicine, College of Health Sciences, Addis Ababa University, Addis Ababa, Ethiopia; Eck Institute for Global Health, Department of Biological Sciences, 319 Galvin Life Sciences, University of Notre Dame, South Bend, IN 46556, USA; School of Public Health, College of Health Sciences, Addis Ababa University, Addis Ababa, Ethiopia; Program in Public Health, College of Health Sciences, University of California at Irvine, Irvine, CA 92697, USA; School of Public Health, College of Health Sciences, Addis Ababa University, Addis Ababa, Ethiopia

**Keywords:** Africa, inequality, insecticide-treated net use, Kenya, pregnant women, socio-economic, subnational region

## Abstract

**Background:**

Insecticide-treated net (ITN) use is among the most recommended strategies to prevent malaria in pregnancy. We analysed the regional and socio-economic patterns of ITN use among pregnant women in Kenya using data from the 2003, 2008 and 2014 Kenyan Demographic and Health Surveys (KDHSs).

**Methods:**

Inequality was assessed using four dimensions: economic status, education, place of residence and region. Both relative and absolute summary measures were applied. In addition, simple and complex summary measures, i.e. difference, population attributable fraction, population attributable risk and ratio were considered based on the number of subgroups in each variable.

**Results:**

There was overt inequality in the use of ITNs among pregnant women, with greater use among the better-off group in 2003 and 2014. Greater ITN use was also observed among pregnant women with a higher level of education. Pregnant women from urban settings tended to use ITNs (slept under a net the night before the survey) more than their rural counterparts in the 2003 KDHS. There were significant regional variations across the three surveys in all inequality summary measures, except ratio in the 2014 survey.

**Conclusions:**

Significant inequality in ITN use among pregnant women was observed at a macro scale.

## Introduction

Malaria is one of the most important public health problems in the world. The disease is particularly dangerous for pregnant women and their foetuses. Each year approximately 19–25 million women in malaria-endemic areas of Africa become pregnant and are at risk of being infected by *Plasmodium* parasites.^1,2^

Malaria in pregnancy can have serious consequences for the health of the mother and the foetus. It is associated to maternal anaemia and maternal mortality^3^ and can cause miscarriage, stillbirth, premature birth, intrauterine growth retardation and low birthweight.^4^ According to the global Malaria and Pregnancy Network report, malaria was associated with approximately 15% of all maternal anaemia cases and 35% of low birthweight babies.^5^ The consequences of malaria during pregnancy vary by transmission intensity (which varies geographically), level of immunity and other factors.^6^ Symptomatic infections are more common in low malaria transmission settings, where acquired immunity is low, and among primigravida women.^7,8^

Insecticide-treated nets (ITNs) are a highly cost-effective public health intervention for prevention of malaria during pregnancy, especially in sub-Saharan Africa.^4^ In Malawi and Burkina Faso, ITN utilization during pregnancy was associated with a decrease in placental malaria and low birthweight.^9,10^ Several studies have described the association between adequate knowledge and attitudes toward ITN utilization. These studies have shown that age, marital status, income, local intensity of malaria transmission and other factors influence the utilization of ITNs.^1,4,9,11,12^ ITN use among pregnant women is often suboptimal and further work is needed to understand and overcome barriers to their use.

**Figure 1.**
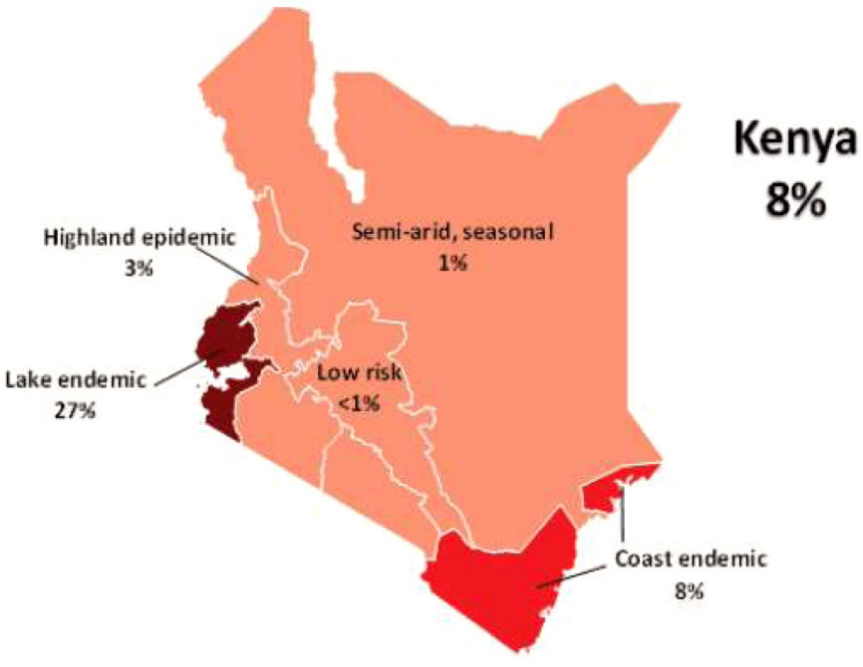
Malaria prevalence in Kenya by zone.

Malaria remains a major public health problem in Kenya. There are an estimated 3.5 million new clinical cases and 10 700 deaths each year, and western Kenya has a higher risk of malaria.^13^ The disease is heterogeneously distributed across the landscape as a consequence of variations in altitude, rainfall and temperature. The national health authorities have defined geographic areas by their relative malaria transmission intensity into four main categories: endemic areas, which include areas around Lake Victoria in western Kenya and in the coastal regions; seasonal malaria transmission areas, which include arid and semi-arid areas of the northern and southeastern parts of the country; highland epidemic-prone areas, which include the western highland part of Kenya; and low-risk malaria areas, which include the central highlands of Kenya and Nairobi^4,14–17^ (Figure 1). Approximately 40% of the total population lives in low malaria risk areas, 27.4% live in endemic zones, 19.8% live in epidemic-prone areas and 12.6% live in seasonal transmission areas.^18^ Deployment of public health interventions is tailored according to these different epidemiological zones, with the endemic and highland epidemic zones being the primary focus areas for ITN distribution.^19^

Malaria prevention in pregnancy includes the use of intermittent preventive treatment (IPT) and provision of ITNs, which have been integrated into routine antenatal care (ANC).^20^ However, malaria prevalence among pregnant women remains high in the country, resulting in significant morbidity and mortality. One likely reason is that ITN access and use remain inadequate. Until 2000, the only source of ITNs was the private retail sector and non-governmental organizations.^21^ Since then, several initiatives have been geared towards increasing access and reducing barriers to access and the use of ITNs.^22–28^ Nevertheless, disparities remain.^29–31^

In view of this persisting problem, the goal of this research was to examine the regional and socio-economic inequities in ITN use among pregnant women based on the 2003, 2008 and 2014 Kenyan Demographic Health Surveys (KDHSs). The evidence generated from our study contributes to the national efforts to achieve the Sustainable Development Goals (SDGs), which includes goals of universal coverage and ultimately reduced inequities.

## Methods

### Study setting

Kenya is located on the East African coast, along the equator. The capital is Nairobi (which has its own province) and the nation has seven other provinces: Central, Coast, Eastern, North Eastern, Rift Valley, Western and North Eastern, covering 580 370 km^2^.^32^ Its climate varies from tropical in the south, west and central regions to arid and semi-arid in the north and northeast. The mean annual temperature is 80°F and most of its inland area is dry.^34^ The estimated current population of Kenya is 54 985 698.^35,36^ About 14 million people live in malaria-endemic areas and 17 million reside in epidemic and seasonal malaria areas.^37^

### Data sources

This study is based on data from the 2003, 2008, and 2014 KDHSs, accessed through the World Health Organization's Health Equity Assessment Toolkit (HEAT).^38^ The 2003 KDHS survey included 8195 women ages 15–49 y and 3 578 men ages 15–54 y.^39^ The 2008 KDHS included 8444 women ages 15–49 y in all households and 3465 men ages 15–54 y in half of households.^40^ The 2014 KDHS included 31 079 women ages 15–49 y and 12 819 men ages 15–54 y.^41^ Surveys were implemented by the Central Bureau of Statistics in collaboration with the Ministry of Health in accordance with relevant ethical guidelines and regulations.^39^

### Variables and measurements

The variable of interest was the proportion of pregnant women sleeping under an ITN the night preceding the survey according to four dimensions: economic status, education, place of residence and subnational region. The choice of these measures of inequality was based on their hypothesized relevance to ITN use as well as availability among pregnant women.

The economic status was measured in terms of the wealth index, which is a composite measure of a household's cumulative living standards. The wealth index is calculated based on water and sanitation facilities; radio or television ownership; materials used for the house floor, roof and walls; car, bicycle or motorcycle ownership; and electricity. Principal components analysis was used to reduce the data to the wealth index,^42^ which was then classified into five categories: poorest, poor, middle, rich and richest.^43^ Educational status was categorized as no education, primary, secondary or higher level. Place of residence was categorized as urban or rural based on official national designation. The variables were measured in the same way in the three surveys.

### Statistical analysis

Summary measures were computed for the disaggregated data from each survey using the HEAT version 3.1 software.^38^ Four summary measures were used to assess inequality: difference (D), population attributable fraction (PAF), population attributable risk (PAR) and ratio (R). For an appropriate description of the outcome of interest, we incorporated R and PAF as a relative measure, with D and PAR as absolute summary measures. Since our variables have only two or more than two subgroups, using both simple measures (D and R) and complex measures(PAR and PAF) was found to be appropriate.^44^

### Description of summary measures

The D for education was calculated as the percentage of pregnant women sleeping under an ITN in the advantaged group (secondary education or higher) minus the percentage in the disadvantaged group (uneducated). For economic status, D was calculated as the percentage of pregnant women sleeping under an ITN in the advantaged group (richest) minus the percentage in the disadvantaged group (poorest). For place of residence, D was calculated as the difference in ITN utilization between rural and urban dwellers. For subnational regions, D indicates the difference between a region with the lowest estimate of pregnant women sleeping under ITNs and the region with the highest estimate of pregnant women sleeping under ITNs. The R was calculated in a similar way as D, except that division was used as the mathematical operation instead of subtraction. PAR was calculated as the difference between the estimate of pregnant women sleeping under ITNs for the advantaged subgroups (as described above) and the national average for the proportion of pregnant women sleeping under ITNs. PAF was calculated by dividing the PAR by the national average (μ) and multiplying the fraction by 100, i.e. [PAF=(PAR/μ)×100].

### Ethical considerations

Survey procedures and resulting data were reviewed and approved by the Inner City Fund (ICF) institutional review board (IRB) to ensure that the survey complied with the US Department of Health and Human Services regulations for the protection of human subjects. Surveys were also reviewed and approved by an in-country IRB to ensure compliance with national laws and norms.^45^ The analyses were carried out on publicly available anonymized data and we did not request ethical approval.

## Results

As shown in Table 1, regions in Kenya had differences in the distribution of wealth and educational attainment during the three KDHSs. In all three, most of the population living in Nairobi (malaria free) belonged to the fourth and highest wealth quintiles, whereas most people in the northeastern region (seasonal malaria) belonged to the lowest and second wealth quintiles. In malaria-stable zones, like Nyanza and the western region, there was a similar pattern of wealth distribution across surveys, where a majority of the population belonged to the second and middle wealth quintiles. There was also variation across regions with regard to educational attainment. During the 2014 KDHS, the northeastern, eastern, Rift Valley, western and Nyanza regions had the lowest educational attainment, whereas more than half the population of Nairobi and the central region had completed primary school or above. In all regions except the central region, more males completed primary school or above than females.

**Table 1.** Wealth quintile and educational attainment distribution across subnational regions in Kenya (DHS 2003, 2008 and 2014)

Year	Dimension	Coast		North Eastern		Eastern		Central		Rift Valley		Western		Nyanza		Nairobi	
2014	Wealth quintile Lowest Second Middle Fourth Highest	40.0 10.4 10.6 14.5 24.5		72.9 4.5 4.5 9.0 9.1		19.5 27.7 22.4 19.7 10.7		2.4 12.0 21.9 32.0 31.7		26.5 20.2 19.7 19.1 14.5		12.3 30.9 33.8 16.8 6.2		16.6 31.2 23.8 17.5 10.9		0.2 0.8 6.0 25.6 67.4	
2008	Wealth quintile Lowest Second Middle Fourth Highest	26.3 12.4 9.4 16.2 35.7		75.9 5.6 5.5 4.9 8.0		16.7 22.3 27.6 26.3 7.1		2.2 11.1 29.2 36.0 21.4		28.2 19.2 16.1 17.8 18.7		17.9 33.0 25.1 18.8 5.2		17.6 28.4 23.9 17.7 12.3		0.0 0.0 0.2 4.2 95.5	
2003	Wealth quintile Lowest Second Middle Fourth Highest	22.7 10.6 13.6 15.7 37.4		71.6 10.8 9.3 4.6 3.7		12.3 20.7 27.3 27.2 12.4		0.9 12.7 27.7 36.0 22.7		22.1 16.5 14.6 22.7 24.1		20.3 30.2 27.6 14.2 7.7		23.7 29.6 20.1 12.9 13.6		0.0 0.0 0.0 2.9 97.1	
2014	Educational attainment	Male	Female	Male	Female	Male	Female	Male	Female	Male	Female	Male	Female	Male	Female	Male	Female
	No education Some primary Completed primary Some secondary Completed secondary More than secondary	15.9 38.2 17.1 8.1 13.7 6.5	26.5 39.8 14.3 6.3 9.0 3.8	49.2 35.0 4.8 4.8 3.0 2.8	69.0 23.4 2.3 2.1 1.5 1.1	8.5 49.0 17.5 9.0 10.2 5.8	14.4 45.8 19.0 9.0 6.9 4.8	3.8 36.3 19.1 12.7 18.9 9.2	7.2 35.0 21.1 13.1 14.2 9.3	13.8 44.4 14.7 8.2 11.2 7.6	18.2 44.0 14.0 8.8 8.4 6.4	8.8 54.7 11.8 11.3 8.5 4.7	12.5 55.2 12.0 10.8 5.3 4.1	10.7 45.3 14.8 11.1 10.6 7.2	13.7 49.1 14.9 10.2 7.6 4.2	3.1 21.9 17.0 9.1 26.4 22.0	4.5 24.0 18.3 11.3 22.7 18.9
2008	Educational attainment No education Some primary Completed primary Some secondary Completed secondary More than secondary	17.6 34.1 19.6 7.5 14.7 6.4	33.1 36.8 13.1 5.3 8.5 3.2	49.1 35.7 6.6 3.1 3.2 2.3	69.6 23.9 2.4 1.4 1.6 1.0	14.2 48.6 17.7 7.2 9.2 2.8	20.8 45.9 17.3 6.5 6.6 2.7	5.3 41.8 23.7 9.1 14.7 5.3	10.9 38.2 25.3 10.0 11.2 4.2	16.5 43.4 15.4 6.6 13.5 4.4	21.5 43.7 16.9 5.7 9.0 3.1	9.9 53.6 15.3 10.3 8.5 2.3	14.2 55.4 14.2 7.3 6.9 1.6	8.9 48.4 17.6 9.7 9.3 5.9	13.4 49.3 17.4 9.3 6.4 4.0	4.6 16.9 12.7 7.4 27.0 31.1	6.1 20.4 17.8 9.6 20.5 25.3
2003	Educational attainment No education Some primary Completed primary Some secondary Completed secondary More than secondary	23.5 39.4 17.9 5.3 10.2 3.0	37.8 36.7 13.0 3.5 5.8 2.7	65.2 28.3 2.5 1.1 2.3 0.8	86.8 11.9 0.5 0.3 0.1 0.2	14.3 53.9 15.7 4.8 7.2 3.6	21.2 49.2 17.1 4.5 5.6 2.3	6.8 44.6 20.8 8.3 12.4 6.4	12.0 43.1 20.5 8.2 10.8 5.0	22.7 44.4 14.4 4.6 9.1 4.3	28.6 43.4 13.8 5.3 5.8 2.6	11.4 56.8 12.6 7.6 7.2 3.5	18.2 55.1 12.1 7.9 4.2 1.6	10.3 55.0 13.3 8.5 8.9 3.7	18.3 54.9 12.2 8.7 4.0 1.8	7.3 21.2 16.0 9.4 28.9 16.4	10.0 22.9 20.4 8.9 21.8 15.4

### ITN utilization by wealth index and education

The proportion of pregnant women who slept under ITNs the night before the survey increased sharply from 2003 to 2008. However, the increase was heterogeneous.

**Figure 2.**
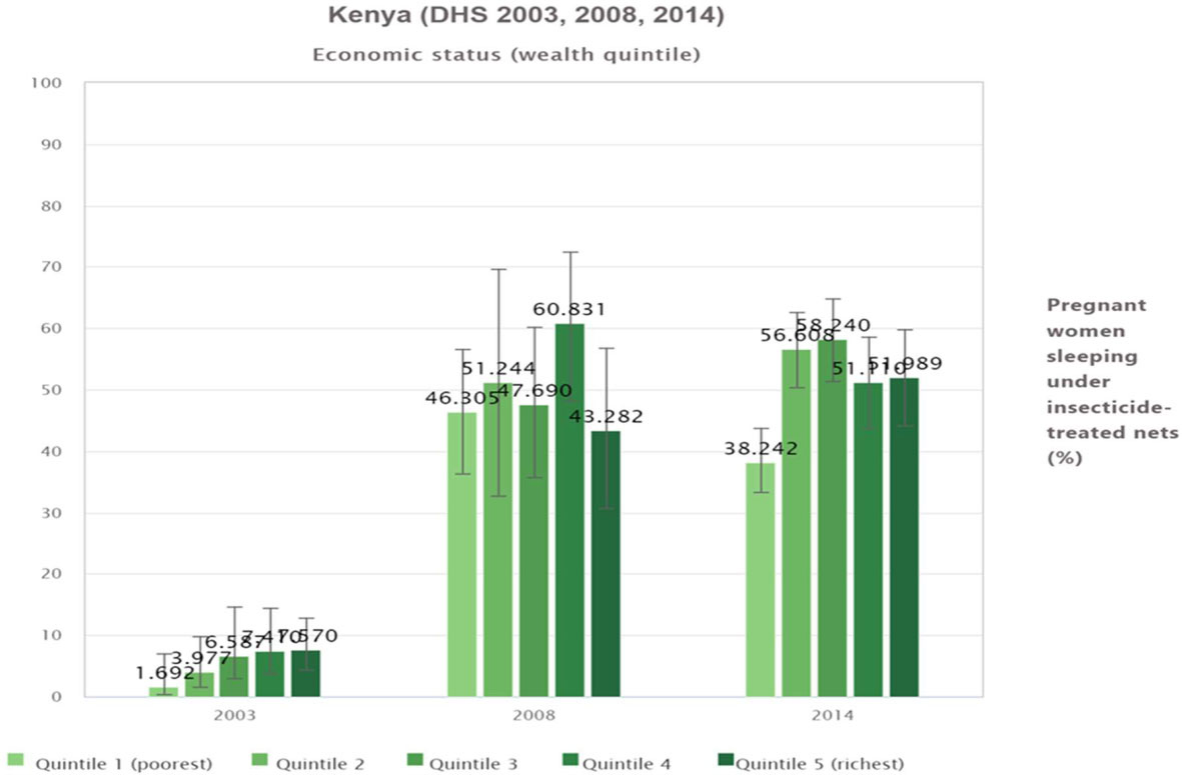
Proportion of pregnant women sleeping under ITNs by wealth quintile in Kenya (DHS 2003, 2008 and 2014).

The poorest group (quintile 1) increased >27-fold (from 1.7 to 46.3%), while the wealthiest group (quintile 5) increased by 5.7-fold (Figure 2). Conversely, data from pregnant women in the third (58.2%) and second (56.6%) quintiles showed better ITN utilization compared with other quintile groups in 2014. Overall, ITN utilization consistently improved from 2003 to 2014 in the second and third quintile groups while the increment was low among the poorest women in 2014 (Figure 2).

**Figure 3.**
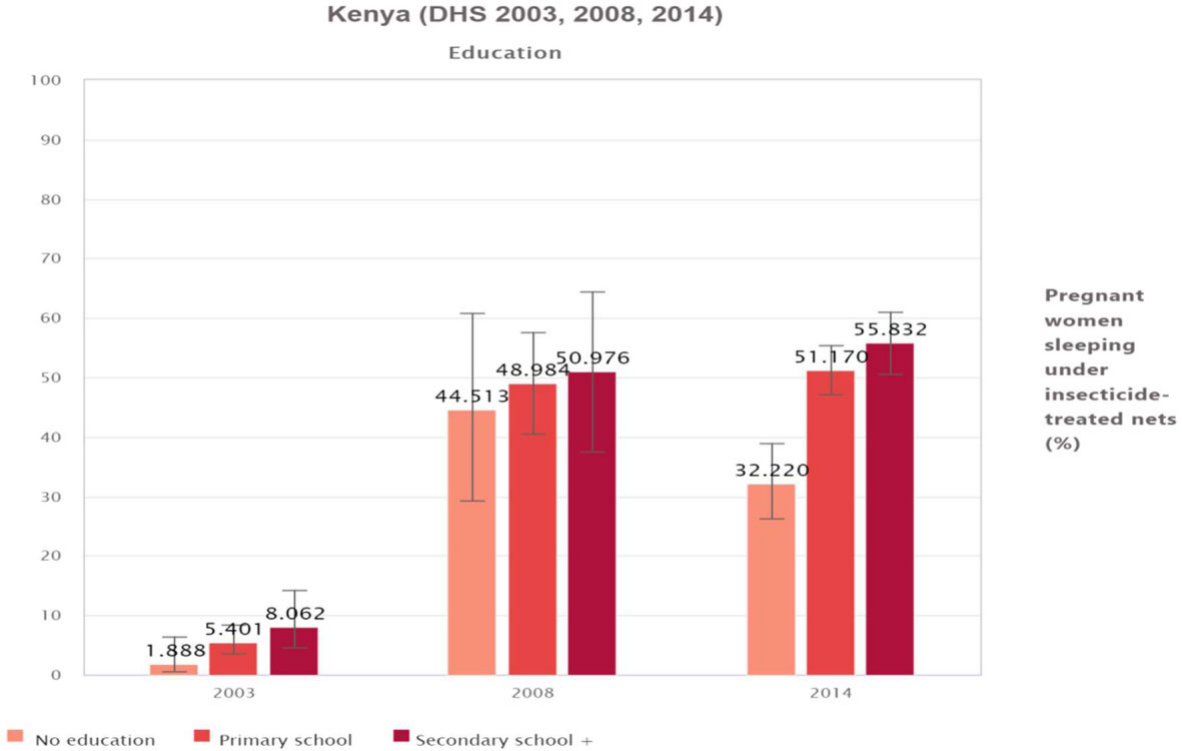
Percentage of pregnant women sleeping under ITNs by education in Kenya (DHS 2003, 2008 and 2014).

Similarly, ITN use varied across educational status. The utilization was higher among educated pregnant women (above secondary school level), with consistent increments across the years: 8.1% in 2003, 51% in 2008 and 55.8% in 2014. Overall, ITN utilization substantially increased from 2003 to 2014, with a sharp increase from 2003 to 2008. Comparatively, the difference in ITN utilization between uneducated and educated groups was largest in 2014. The most disadvantaged group over the years was that of illiterate pregnant women (Figure 3).

### ITN utilization by residence (urban vs rural settings) and subnational regions

**Figure 4.**
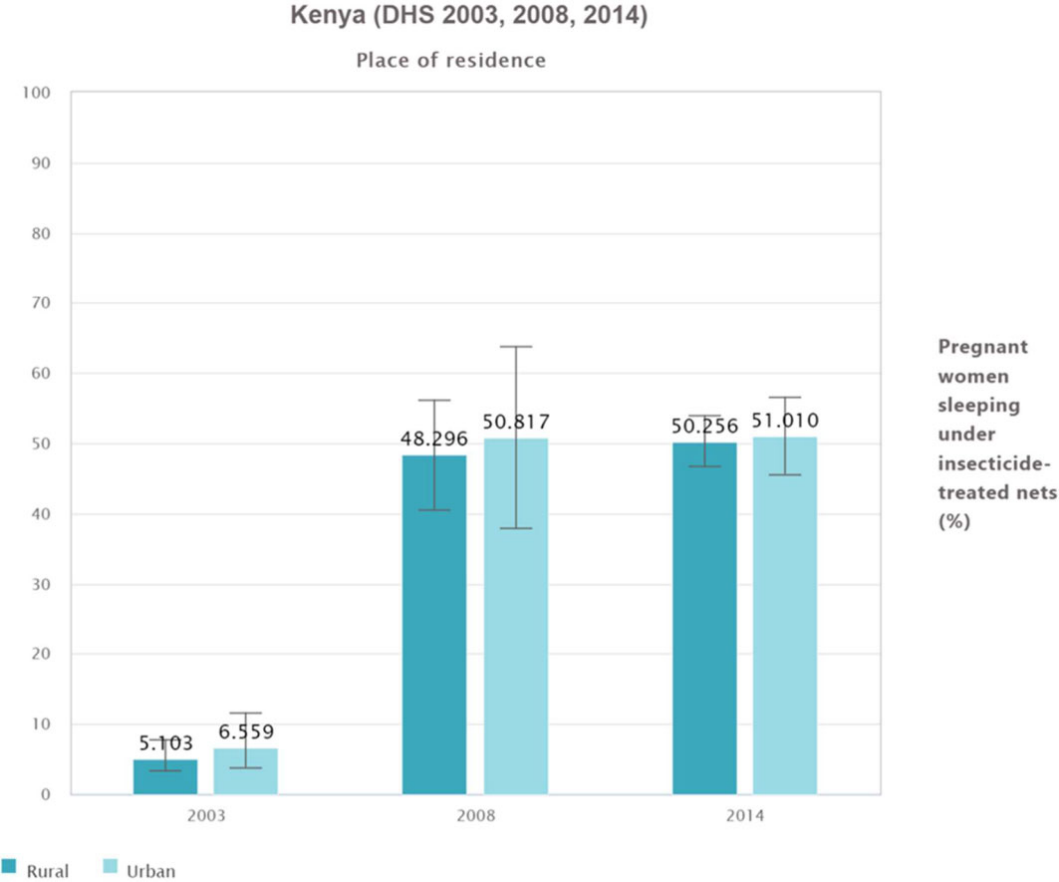
Pregnant women sleeping under ITNs by place of residence in Kenya (DHS 2003, 2008 and 2014).

Comparable ITN use was observed between pregnant women in urban and rural parts of Kenya across the three surveys. Pregnant women in urban areas used ITNs more than their rural counterparts, although the difference was minimal and there was an increasing trend of ITN utilization over the 3 y for both groups (Figure 4).

Utilization of ITNs varied across subnational regions across surveys. In 2003, the proportion of women who slept under ITNs was 11.9% in central Kenya and 10.5% in Nyanza, and these were among the highest ITN utilizers when compared with other subregions. In 2008, the proportion of women who slept under an ITN was 69.3%, 60.5% and 64% in the western, northeastern and coastal subregions, respectively, and these were among the highest ITN utilizers. In 2014 (Table 2), the proportion of ITN utilizers varied slightly across subregions—71.2% in Nyanza, 63.1% in the coastal subregion and 66.7% in western Kenya—and were relatively higher when compared with central Kenya (36%), Nairobi (43.3%) and the Rift Valley (40.6%). (Table 2). Unlike the high-prevalence regions such as the Coast and Western provinces, the proportion of pregnant women who slept under ITNs the night before the survey in Nyanza increased over the three KDHSs, as well as provinces in the highlands (malaria epidemic prone).

**Table 2.** Proportion of pregnant women sleeping under ITNs in Kenya across subnational region (2003, 2008 and 2014 KDHS)

		Estimate (95% CI)		
Dimension of inequality	Subgroup	2003	2008	2014
Subnational region	Nairobi Central Coast Eastern Nyanza Rift Valley Western North eastern	2 (0.8 to 12.2) 11.9 (5.8 to 22.9) 6.0 (2.4 to 14.4) 4.8 (1.6 to 13.1) 10.5 (5.4 to 19.3) 1.0 (0.1 to 6.7) 4.5 (1.5 to 12.6) 6.8 (1.8 to 22.2)	45.8 (26.5 to 66.5) 26.1 (14.0 to 43.3) 64.0 (52.9 to 73.8) 53.6 (39.4 to 67.2) 57.6 (48.7 to 66.0) 29.5 (17.0 to 46.2) 69.3 (49.6 to 83.7) 60.5 (48.2 to 71.6)	43.3 (31.1 to 56.5) 36.0 (27.3 to 45.8) 63.1 (55.0 to 70.5) 49.8 (40.3 to 59.3) 71.2 (63.1 to 78.1) 40.6 (35.7 to 45.7) 66.7 (58.2 to 74.2) 43.4 (31.8 to 55.8)

### Variation in ITN use among pregnant women by indicators of inequality

Considerable variation in ITN use was observed across all indicators of inequality in all summary measures (D, PAR, PAF and R). In the 2003 and 2014 surveys, the absolute difference across wealth quintiles confirmed significant economic inequality, with higher ITN utilization among the richest subgroup when compared with the poorest subgroup (D=5.9 [95% confidence interval {CI} 1.2 to 10.6] in 2003 and 13.7 [95% CI 4.2 to 23.3] in 2014). In contrast, the highest ITN utilization was seen among the poorest subgroup (D=−3.0 [95% CI −19.8 to 13.7]) in 2008, although the difference was not statistically significant. A decreasing trend in inequality based on economic status was observed from 2003 to 2014. Unlike the 2003 and 2014 surveys, the inequality across all dimensions was insignificant in 2008 and favoured the disadvantaged group (R=0.9 [95% CI 0.6 to 1.3]). Nonetheless, overall the findings indicate that a change in economic status was associated with ITN utilization.

Regarding educational status, in 2003 and 2014, ITN utilization was significantly higher among the educated subgroup (D=6.2 [95% CI 1.1 to 11.3] and 23.6 [95% CI 15.4 to 31.8], respectively). The PAF and R showed that inequality by educational status was substantially reduced from 2003 to 2014. However, the inequality in ITN utilization remained comparatively high in both the 2003 and 2014 surveys in all inequality dimensions. The 2008 survey was showed the least inequality, yet showed disparities in the educational status.

Overall, urban–rural disparities in ITN use existed throughout the surveys. In 2003 and 2008, relatively more pregnant women slept under an ITN in urban areas than their rural counterparts, while in the 2014 survey, rural dwellers did so, although the difference was not significant.

Statistically significant spatial variations in ITN use were also observed across surveys. Overall, the inequality across subnational regions was markedly reduced in 2014 compared with the preceding years in all summary measures (Table 3).

**Table 3.** Magnitude of socio-economic and subregional inequality in pregnant women sleeping under ITNs in Kenya

		Estimate (95% CI)		
Dimension of inequality	Measurement	2003	2008	2014
Economic status	D PAF PAR R	5.9 (1.2 to 10.6) 39.9 (−11.8 to 91.6) 2.2 (−0.6 to 5.0) 4.5 (−2.4 to 11.3)	−3.0 (−19.8 to 13.7) 0.0 (−14.9 to 14.9) 0.0 (−7.3 to 7.3) 0.9 (0.6 to 1.3)	13.7 (4.2 to 23.3) 2.8 (−5.0 to 10.7) 1.4 (−2.5 to 5.4) 1.4 (1.1 to 1.6)
Education	D	6.2 (1.1 to 11.3)	6.5 (−14.8 to 27.7)	23.6 (15.4 to 31.8)
	PAF	48.9 (−18.8 to 116.7)	4.2 (−17.6 to 26.1)	10.4 (−0.7 to 21.6)
	PAR	2.6 (−1.0 to 6.3)	2.1 (−8.6 to 12.7)	5.3 (−0.4 to 10.9)
	R	4.3(−1.4 to 10.0)	1.1(0.6 to 1.7)	1.7 (1.4 to 2.1)
Place of residence	D PAF PAR R	1.5(−2.9 to 5.8) 21.2 (3.1 to 39.2) 1.1(0.2 to 2.1) 1.3(0.4 to 2.2)	2.5(−12.9 to 17.9) 3.9 (−0.8 to 8.6) 1.9 (−0.4 to 4.2) 1.1 (0.7 to 1.4)	0.8 (−5.9 to 7.4) 0.9 (−2.6 to 4.5) 0.5 (−1.3 to 2.3) 1.0 (0.9 to 1.1)
Subnational region	D PAF PAR R	10.9(2.5 to 19.3) 119.7 (79.5 to 159.8) 6.5 (4.3 to 8.7) 12.0 (−12.6 to 36.6)	43.2 (20.2 to 66.1) 41.6 (15.1 to 68.1) 20.3 (7.4 to 33.3) 2.7 (1.0 to 4.3)	35.1 (23.2 to 47.1) 40.8 (27.9 to 53.6) 20.6 (14.1 to 27.1) 2.0 (1.4 to 2.5)

## Discussion

This study examined regional and socio-economic inequalities in the use of ITNs among pregnant women in Kenya based on three DHSs. The goal of this work was to describe patterns of ITN use among pregnant women in Kenya over time so that appropriate strategies, if needed, could be developed to enhance the use of ITNs, since it is one of the most effective and affordable strategies to prevent malaria in pregnancy.^10,11^

Results showed that ITN use was more common among wealthy pregnant women. This pattern may be related to a general lack of access to ITNs and a lack of equity in the national healthcare system to ensure fair access to health interventions, hampering efforts to achieve the SDGs. Several small-scale studies in Kenya found that access to ITNs is hindered by their cost.^3^ The medical costs of malaria disease treatment could exacerbate the socio-economic situation and have a profound impact on household economies.^10^ A study in the Kilifi district reported that 85% of non-ITN use in pregnancy was the result of a lack of money.^4^ The same disparity was observed in Senegal, where the poorest quintile group also had the lowest use of ITNs (n=12), and Uganda, where the high cost of ITNs affected malaria prevention measures in pregnancy.^46^

It is worth noting that low utilization, does not necessarily imply a lack of access to ITNs. Household possession of ITNs does not necessarily imply utilization by pregnant women or other vulnerable segments of the population, especially if ITNs are not sufficient to cover the whole family. This calls for a targeted national campaign to ensure ITN access and utilization among pregnant women as well as to increase use awareness.

Interestingly, in 2008 there were no significant disparities observed in ITN utilization by economic status among pregnant women. This may be related to the government deployment of ITNs free of charge towards the end of 2006. This provided the opportunity to reduce the pre-existing differences in ITN ownership between the upper and lower economic classes.^4^ Towards 2014, however, cost again became a barrier to ITN access, especially for the lower income groups.^31^

Higher ITN utilization was observed in women with a higher level of education. This finding confirms previous data from Kenya, although a different result was observed in The Gambia, where villagers with no education used ITNs more frequently than those with some form of formal education.^4,11^ Similarly, the 2015 Ghana DHS showed that more uneducated pregnant women used ITNs (49.6% vs 43.5%) when compared with educated women up to middle school level.^47^ The same study reported that most respondents had knowledge about the mode of transmission of malaria and ITN use to prevent malaria with a ‘know–do gap’. In addition to universal education, sociocultural factors are a key target for behavioural interventions aimed at improving adherence to malaria control programs.^4^

In this study, more pregnant women in urban areas slept under ITNs than in rural areas, similar to what was found in Equatorial Guinea.^48^ In Nigeria, greater ITN ownership was found among urban inhabitants, although ownership of ITNs does not necessarily guarantee higher utilization.^49^

Another important finding that emerged in this study is the spatial variability observed in ITN use among subnational regions. In some provinces with a high malaria burden, the proportion of pregnant women who slept under ITNs the night preceding the survey did not show a continuous increment over the three KDHSs. However, it did increase in epidemic-prone areas over the 3 y.

This study is based on the analysis of data from nationally representative samples and we applied methods of measuring inequality that are widely used in the field of measurement of disparities. However, the limitation is that we were unable to explore further the specific causes of disparity.

In conclusion, our study indicated that although most regions experienced an increase in use of ITNs over time, there were marked differences in the use of ITNs by socio-economic status, geographic region and education level. These findings can help relevant stakeholders in targeting demographic groups and specific regions to improve access to and use of ITNs in pregnant women in Kenya.

## Data Availability

The datasets generated and/or analysed during the current study are available in the WHO HEAT version 3.1 software repository (https://whoequity.shinyapps.io/HEAT/).
